# Bone Regenerate Evaluation Methods *

**DOI:** 10.1055/s-0043-1776021

**Published:** 2024-03-21

**Authors:** Gracielle Silva Cardoso, Renato Amorim

**Affiliations:** 1 Serviço de Ortopedia e Traumatologia, Hospital Governador Celso Ramos, Florianópolis, SC, Brasil

**Keywords:** quantitative evaluation, osteogenic distraction, biomechanical phenomena, external fixation, X-ray

## Abstract

Since its introduction by Ilizarov, the distraction osteogenesis technique has been used to treat trauma-related conditions, infections, bone tumors, and congenital diseases, either as methods of bone transport or elongation. One of the major dilemmas for the orthopedic surgeon who performs osteogenic distraction is establishing a reproducible method of assessing the progression of the osteogenesis, enabling the early detection of regenerate failures, in order to effectively interfere during treatment, and to determine the appropriate time to remove the external fixator. Several quantitative monitoring methods to evaluate the structural recovery and biomechanical properties of the bone regenerate at different stages, as well as the bone healing process, are under study. These methods can reveal data on bone metabolism, stiffness, bone mineral content, and bone mineral density. The present review comprehensively summarizes the most recent techniques to assess bone healing during osteogenic distraction, including conventional radiography and pixel values in digital radiology, ultrasonography, bone densitometry and scintigraphy, quantitative computed tomography, biomechanical evaluation, biochemical markers, and mathematical models. We believe it is crucial to know the different methods currently available, and we understand that using several monitoring methods simultaneously can be an ideal solution, pointing to a future direction in the follow-up of osteogenic distraction.

## Introduction

Since its introduction by Ilizarov, the distraction osteogenesis technique has been used to treat trauma-related conditions, infections, bone tumors, and congenital diseases, either as a bone transport or elongation method. The success of the distraction osteogenesis technique relies upon a number of factors, including low-energy osteotomy, stable fixation, acceptable latency period, adequate distraction rhythm, and functional limb maintenance during distraction. 1 The location of the osteotomy also influences the quality of the regenerate; metaphyseal osteotomies are more likely to lead to sufficient bone callus formation. The latency period between the osteotomy and the beginning of the distraction ranges from 5 to 7 days, that is, the time required for the formation and organization of the hematoma to maximize the development of a regenerate with proper vascularization. Most authors recommend a distraction rate of 0.25 mm 4 times a day. 1

Careful anamnesis and physical examination are required before starting the osteogenic distraction process. It is critical to identify some risk factors that may disturb the formation of the regenerate, such as concomitant diseases, drug use and smoking, age, malnutrition, diabetes mellitus, chronic use of non-steroidal anti-inflammatory drugs, the etiology of the abnormality, previous surgeries at the osteotomy sites, tumors, and vascularization changes in the surrounding tissues. 1 2 3

One of the major dilemmas for the orthopedic surgeon who performs osteogenic distraction is establishing a reproducible method of assessing the progression of the , enabling the early detection of regenerate failures, in order to effectively interfere during treatment, and to determine the appropriate time to remove the external fixator. 4

## Regenerate assessment and monitoring

### Radiography

There are several ways to monitor the formation of the bone regenerate. Plain radiography is the most widely used method for evaluation and monitoring, 1 and it is the most readily-available and clinically-accessible test. 3 5 As such, plain radiography is the most cost-effective imaging method to monitor all aspects of bone regeneration, 6 7 even though it depends on the experience of the professional in charge of the evaluation. 1

The major disadvantage of radiography compared with other methods in the follow-up of patients undergoing bone lengthening procedures is its inability to detect the presence of a new regenerate until the deposition of a considerable amount of calcium. 3 Biologically, calcification lags to osteoid formation, and a few weeks may pass before radiography shows any evidence of bone response to stretching. 8

The success of osteogenic distraction depends on serial radiographic evaluation, which is essential to guide decisions, such as removing external fixators and changing the distraction rate. The observation of three to four healed cortices on anteroposterior and lateral radiographs is commonly used as an endpoint for healing. 9 10 While useful, there is much disagreement among practitioners regarding this this method, with reported interobserver rates lower than 0.5. 11 Plain radiography and clinical examination usually back the decision to remove the fixator; however, fracture rates ranging from 30% to 50% have been reported when using these criteria. 12 13 14 The observation of 2 mm of cortex with a density similar to that of normal bone and three cortices on radiography define adequate corticalization. For elongations greater than 10 cm or 50% of the original tibial length, the presence of 3 cortices seems inadequate, and delaying the time to remove the fixator until the fourth cortex is also well formed is advisable to prevent the delayed subsidence of the tibia. 15

Shyam et al. 15 described the callus diameter ratio, calculated by the average anteroposterior and lateral diameters of the callus divided by the average diameters of the proximal and distal ends of the corticotomy. Mamada et al. 16 reported a significant increase in the fracture rate when this ratio was lower than 80%.

Traditional radiographic techniques enable the qualitative assessment of new bone formation; however, the formation of new bone has never been properly quantified. The lack of an objective measurement method may result in high intraobserver and interobserver errors in radiographic subjective measurements. 17

Starr et al. 18 evaluated the commonly-cited criterion of the presence of 3 of the 4 continuous cortices with at least 2 mm in thickness on anteroposterior and lateral radiographs. These authors 18 reported low mean kappa reliability coefficients for intraobserver (0.290) and interobserver (0.127) responses, showing that the assessment of the number of cortices by itself is a poor indicator of the moment to remove the fixator and also showing its insufficiency as an isolated method.

Eyres et al. 19 reported that although ultrasonography and bone densitometry using dual-energy X-ray absorptiometry (DEXA) provides valuable information on the distribution and amount of new bone formed during distraction osteogenesis, high-resolution radiography helps in the detection of small cortical defects not identified by other imaging techniques.

### Pixel value in digital radiology

Traditional radiographic techniques enable the qualitative assessment of new bone formation, but only digital radiology can quantify it. 8 20 The quantitative methods include quantitative computed tomography (QTC), 21 22 quantitative technetium scintigraphy, 20 and DEXA bone densitometry, 19 and they measure the mineralization of the bone regenerate, which correlates with its stiffness; 23 however, they are costly and require the patient to undergo additional imaging tests. 24 The use of pixel value on digital radiographs 14 25 has been proven to be a cost-effective method to measure changes in regenerate mineralization and to provide objective parameters for the decision-making process. 17

The pixel value enables the evaluation of the bone mineral density (BMD), as well as the assessment of the healing of the bone regenerate by comparing its density to that of the adjacent bone. As the density of the bone regenerate increases with healing, its pixel value approaches that of the adjacent normal bone. 26.27

Gray value (GV) is another indicator to assess bone healing. A grayscale image is a data matrix that represents a specific range of brightness values in which 0 means black and 255 refers to white. In a grayscale image, the portion with high brightness represents the object with higher density or thickness; the part with low brightness transmission represents the object with lower density or thickness. 28

Singh et al. 6 serially evaluated the pixel values from regenerated and adjacent bone segments during bone lengthening in achondroplastic patients. Next, they calculated the pixel value ratio (pixel ratio = [(average pixel value from the proximal segment + average pixel value from the distal segment)/2]/average pixel value from the new bone formation). Several authors have shown that patients with regenerate fractures present pixel value ratios lower than 0.8 at the time of fixator removal. 5 14 15 17 Pixel value ratios can be used as an adjunct to the digital radiological assessment to help detect early healing disorders and customize weight bearing. These values may also be an objective guide for fixator removal. However, this method does not directly measure the stiffness of the bone regenerate. 4 6

The pixel value technique using digital radiographs minimizes the variation in intra- and interobserver responses observed with plain radiographs; 1 5 in addition, it is a reliable, available, and low-cost method to assess the maturation of the regenerate. 4 17 26 However, as a digital radiology imaging-based mathematical method, the pixel value technique has limitations similar to those of conventional radiology to evaluate the initial regenerate. 26

### Ultrasound

The principle of using ultrasonography for regenerate evaluation is that broadband ultrasonic attenuation signals can assess BMD changes. The ultrasound passing through body tissue suffers attenuation; the amount of attenuation relates to tissue characteristics. The velocity of the ultrasonic waves and the amplitude of the attenuation through bone tissue enable the calculation of the bone mineral content (BMC) and the determination of bone structure and strength. 29 The inability of ultrasound to penetrate cortical bone limits the ultrasonographic assessment of normal mature bone. However, high-resolution linear ultrasound can evaluate new bone with incomplete remodeling and calcification. 1 30 Contrast-enhanced ultrasound (CEUS) can provide an early indication of neovascularization and back the diagnosis of poor bone regeneration. 31 In addition, ultrasound enables the prediction of bone callus formation through the observation of blood flow changes around the new bone, which can compensate for the poor early visualization of the callus on radiography. 32

Ultrasonography is a non-invasive, effective, inexpensive, and ionizing radiation-free method to assess bone healing. It can detect new bone formation four to six weeks before radiography, 26 and it indicates the rate of formation of new bone in the early stages of distraction. 33 Therefore, early assessment is best performed by ultrasound, which enables the detection of unmineralized osteoid and the presence of any defects in the callus. 8

However, Eyres et al. 19 observed that ultrasonography does not detect alterations in the medullary region of the bone after corticalization, even when using a 5-MHz frequency probe. Ultrasound helps to identify defects in the corticalization of the regenerate not recognized by DEXA or radiography. Nevertheless, it is an examiner-dependent method subject to the surgeon's experience and familiarity. In addition, it does not enable the evaluation of bone alignment, and it presents limitations regarding the evaluation of the final stages of osteogenic distraction, with a small role in the decision to remove the external fixator. 26

### Bone densitometry by DEXA

Bone densitometry by DEXA uses X-ray sources to emit two different radiation energies. It enables the measurement of energy absorption through bone and soft tissue separately, discarding the influence of soft tissues. As a result, it measures the BMC and the BMD area (BMDa) and shows changes in bone trabeculae. 34

The ability of bone densitometry to determine the amount and rate of new bone formation is an advantage over other methods such as ultrasound and radiography. Densitometry and ultrasonography can identify new bone within one to two weeks after osteotomy; in contrast, radiography requires four to eight weeks. Bone densitometry also enables the measurement of the alignment and distraction of the limbs through the entire lengthening period. This is an advantage over ultrasonography, which enables the evaluation of the regenerate only during the initial stages of distraction, when corticalization has not yet occurred. 19

Several studies have suggested DEXA scanning as a tool to assess regenerate quality during the distraction phase and to decide the appropriate time for fixator removal. 1 13

Saran and Hamdy 13 used bone densitometry and plain radiography to determine the moment of stabilization of the BMD of the regenerate, and they found rates of fracture and deformity of the bone regenerate after removal of the external fixator of 3.6% and 0% respectively, even with weight bearing as tolerated and no use of immobilization by patients.

Shyam et al. 15 calculated the BMD ratio based on the relationship between the BMDs of the bone regenerate and of the normal bone, and they observed that an index higher than 0.85 would significantly prevent fracture and angulation of the bone regenerate after external fixator removal.

The measurement of cortex mineralization through bone densitometry is an objective method to assess the regenerate, as it enables a quantitative evaluation. However, its limitations include high costs and low relative availability, preventing its clinical applicability on a large scale. 17 26

### Bone scintigraphy

Three-phase bone scintigraphy is a non-invasive method to semiquantitatively assess changes in blood flow, blood distribution, and bone metabolism. The blood supply is considered closely related to the regenerate production capacity in distraction osteogenesis. 35

Kawano et al. 36 assessed whether bone scintigraphy using technetium could help evaluate and predict bone regeneration by comparing clinical indices, such as those of distraction, maturation, and external fixation, with bone scintigraphy data, including the perfusion index, the uptake ratio of the blood-pool image, and the uptake ratio of the delayed image. They concluded that three-phase bone scintigraphy is a reliable method to assess osteogenic distraction compared to clinical indices, especially the uptake ratio of the delayed image, which demonstrated greater predictability.

Despite the predictive potential of bone scintigraphy, few clinical studies have evaluated its use in osteogenic distraction. 36 In addition, its high cost and relatively low availability are limitations to its clinical applicability on a large scale. Moreover, this technique does not enable a concurrent assessment of bone alignment.

### Quantitative Computed Tomography

Quantitative computed tomography measures BMD and evaluates bone alignment and body composition through a special software in a standard scanner. It provides a high-precision, low-error assessment, making it an excellent method to determine BMD changes over time. 26

Quantitative computed tomography relies on the differences in the absorption of ionizing radiation by different tissues, enabling the comparison of attenuation measurements with standard reference values to determine parameters such as the BMC and BMD. 37 In addition, QCT can use three-dimensional images to assess the bone callus, for it enables performance of a finite element analysis to predict bone callus strength, with applications in musculoskeletal research. 26

However, its high cost and radiation dose demand consideration; moreover, currently, it is not widely applicable and available. Further studies are required to address these issues. With the development of QCT, the assessment of the bone regenerate may provide more valuable information, including bone healing monitoring and a prediction of its strength with finite element analysis. 26

### Biomechanical assessment

Measuring changes in bone mechanical properties is the most direct method of assessing the bone healing process. Bone biomechanics is based on engineering mechanics, assessing bone quality per the mechanical properties of bone tissue under external action and the poststress biological effect on bone tissue. 38 The assessment of the mechanical properties of osteogenic distraction and new bone tissue often employs flexion, torsion, tension, and compression tests. 39 40 Bone mechanical parameters, such as flexural and torsional stiffness, help to understand bone healing. 41

The major limitation of biomechanical evaluation for regenerate monitoring is the risk of potential bone damage caused by the stress tests used in the measurement process. As such, the biomechanical evaluation is currently restricted to medical research. 26

Lineham et al. 42 described a potential indirect biomechanical assessment by measuring the deflection of the Kirschner wires used to assemble the circular external fixation during stretching and bone transport. These authors 42 observed that wire deflection was significantly associated with stability determined clinically and radiologically. Although this was a pilot study and the method remains unavailable, developing new in vivo biomechanical measurement devices could help the clinical practice.

### Biochemical markers

Theoretically, an alteration in bone metabolism can lead to subsequent morphological changes. In other words, variations in bone turnover markers (BTMs) levels should occur earlier than identifiable BMD modifications. Therefore, BTMs are a potential new method to assess bone healing and they may be a valuable addition to imaging tests. 26

Several types of BTMs have been identified, 43 including osteocalcin (OC), bone-specific alkaline phosphatase (BSAP), procollagen type I N-terminal propeptide (PINP), and procollagen type I carboxy-terminal propeptide (PICP). Their levels may reflect the biological in vivo activities of osteoblasts and osteoclasts. 44.45

Fink et al. 46 studied the relationship between BTMs and radiographic density during distraction osteogenesis, and they found that serum OC and PICP levels can provide valuable information on bone formation during treatment.

Leung et al. 47 studied a model of osteogenic distraction in goats and found a strong correlation between BSAP activity in plasma and the radiological morphology and biomechanical properties of the new bone. This correlation shows the potential use of BSAP to monitor the process of bone callus change and formation.

Kumar et al. 45 prospectively studied 168 patients with closed tibial fractures treated with locked intramedullary nails, and they demonstrated that BTM (BSAP, OC, and PINP) levels were significantly lower in subjects with late consolidation.

Several bone metabolic markers to monitor bone healing have been reported, some with a high degree of theoretical feasibility. However, further well-designed experimental and clinical studies are still needed to determine the clinical applicability of these biochemical markers in the follow-up and evaluation of the bone regenerate. 26

### Mathematical model

Reina-Romo et al. 48 presented a mathematical model based on a finite element structure to study the spatial and temporal patterns of osteogenic distraction close to the osteotomy site. A distraction rate of 0.3 mm a day resulted in an early increase in mean bone density. Computationally, this lower distraction rate is accompanied by a lower level of mechanical stimulation, which stimulates osteogenesis. In contrast, a distraction rate of 2 mm a day produces nonunion; 49 this finding is consistent with most clinical outcomes that consider that a distraction rate of around 1 mm a day has the best effects on tissue regeneration. 50

Subsequently, Reina-Romo et al. 51 extended the previously-developed differentiation model by incorporating tension-compression asymmetry. The new model considers that bone formation under traction would comprise mainly intramembranous ossification; in contrast, bone formation under a compressive load would consist mostly of endochondral ossification. 52 As such, the mechanical stimulus to activate bone tissue formation would be higher under tension rather than compression. 53

Hence, the biomechanical computational model of the bone transport process based on experimental models could be a useful tool in the follow-up of osteogenic distraction. 54

## Bone regenerate classification

Although radiographs can provide valuable information about the distraction rate and regenerate alignment, qualitative and quantitative assessments of this new bone must be careful until the determination of the reliability and significance of these features. 8

During radiographic evaluation, changes in limb position, beam penetrance, and magnification can significantly alter the image obtained. Observers may interpret the characteristics under scrutiny differently, and the relationship between these characteristics and the outcome is unclear. 8

Several authors have tried to classify bone regeneration; however, with a few exceptions, the reliability and reproducibility of these classification systems have not been tested. 2 8 55 56 Some of these studies also have the disadvantage of presenting relatively small sample sizes and a relatively large number of influencing factors that limit the interpretation of their findings. 49

Table 1 shows a review of the bone regenerate classification systems reported in human and animal studies.

**Table 1:** Overview of bone regenerate classification systems

Author	Year	Characteristics
Catagni 55	1991	Normotrophic bone: first radiodense bone 20 days after corticotomy
		Hypertrophic bone: bone formation before 20 days or bone wider than the osteotomy ends
		Hypotrophic bone: delayed bone formation, 30 days after corticotomy; multiple radiolucency in the regenerate or hourglass-shaped bone
Hamanishi et al. 2	1992	1. External: fusiform regenerate
		2. Straight: homogeneous regenerate as wide as the original bone
		3. Attenuated: regenerate narrower than the original bone
		4. Opposite: regenerate at the end opposite the fixator
		5. Pillar: poor regenerate, only in the central portion
		6. Agenetic: only sparse calcification in the elongated gap
Orbay et al.	1992	Type I: homogeneous new bone joining the two osteotomy ends
		Type II: the osteotomy is covered by a continuous segment of new bone but there is a discontinuity in at least one of its cortices or the bone has an irregular appearance
		Type III: complete radiolucent defect across the site of new bone formation
Minty et al. 56	1994	1: occasional patches of new bone
		2: disorganized callus
		3: regenerate in organized layers
		4: early corticalization
		5: complete bone bridge connecting the two osteotomy ends
Donnan et al. 8	2002	Per the regenerate format (fusiform, contained, opposite, or attenuated)
		Per the regenerate polarity (polarized or non-polarized)
		Per the regenerate consistency (homogeneous, lucent, striated, or speckled)
Li et al. 49	2006	According to shape: based on the width of the callus compared to the original osteotomy osseous site (fusiform, cylindrical, concave, lateral, and central)
		According to type: based on four patterns of osteogenic distraction (sparse, homogeneous, heterogeneous, and transparent) and three densities (low, intermediate, and normal)
Tirawanish and Eamsobhana 58	2018	System matching the diameter and density of the bone regenerate (scores from 2 to 9)
		Part I: diameter – average percentage of the anteroposterior and lateral diameters of the regenerate in relation to the bone diameter at the osteotomy site (classified into groups from 1 to 5)
		Part 2: density – subdivided into 1 (low density), 2 (low intermediate density), 3 (intermediate density), and 4 (high density)

Catagni 55 described his radiological classification of the regenerate during osteogenic distraction with the Ilizarov apparatus based on a clinical experience with more than 800 cases. He classified the bone regenerate as normotrophic, hypertrophic, and hypotrophic, drawing attention to the need for its careful monitoring in search of features that could influence the stretching outcome. Although this classification provided critical insight into problems that may occur during osteogenesis, it was based purely on one observer's experience and does not consider the variability in bone response due to patient age, osteotomy site, or underlying pathology. 8 10 57

Donnan et al. 8 reviewed the existing classification systems and combined the essential characteristics of the other classifications into three groups: form, consistency, and polarity. Based on this new classification, these authors 8 observed moderately good interobserver agreement regarding shape and consistency but only fair agreement regarding polarity. Archer et al. 5 evaluated the inter- and intraobserver agreement of the classification by Donnan et al., 8 and they observed moderate interobserver and good intraobserver reliability.

Li et al. 49 developed a bone regenerate classification system based on the shape of the radiographic callus and the fracture type in different limb-lengthening stages, from osteotomy to distraction, consolidation, and fixator removal. They classified the radiographic features of distraction osteogenesis per shape and type. Shape consisted of callus width compared to the original bone osteotomy site. The type was based on four osteogenic distraction patterns (sparse, homogeneous, heterogeneous, and transparent) and three densities (low, intermediate, and normal).

The grading system developed by Li et al. 49 helps to record and monitor the distraction and healing of the bone regenerate. 4 49 Its reliable interobserver correlation and high level of reproducibility for individual observers makes it useful for the follow-up of distraction osteogenesis. 4 5 49

Using the classification by Li et al., 49 Isaac et al. 3 observed that homogeneous and heterogeneous osteogenic distraction patterns yielded good outcomes, while transparent and sparse patterns led to poor outcomes. As for shape, they noted that fusiform, cylindrical, and lateral shapes yielded good outcomes, while the concave shape led to bad outcomes. Thus, these specific patterns may lead to potentially unsatisfactory outcomes, requiring a therapeutic plan to nullify this effect, such as adjusting the distraction rate and performing distraction-compression or bone grafting.

Tirawanish and Eamsobhana 58 classified callus density into four patterns (three heterogeneous and one homogeneous). Clinically, heterogeneous patterns may alert the surgeon to potential issues, including a high distraction rate, fixator instability, early diastasis of the osteotomy site, or deformity correction. For these authors, the homogenous healing pattern was ideal, while a heterogeneous pattern would have a higher likelihood of a yielding poor outcome. They developed a scoring system applicable to osteogenic distraction in lower-limb lengthening treatments that is used to record and summarize radiographic information; it enables the correlation of the bone callus characteristics, and has the goal of predicting good or bad outcomes. Thus, it is an assessment method to monitor progression and foresee potential problems, enabling the early adjustment of the treatment process if required. A score of 8 or 9, for instance, would indicate a good outcome, while a score lower than 7 would imply a poor outcome. This system has been proven to be reliable and reproducible by experienced and less experienced surgeons.

## Final considerations

There are several methods to perform the quantitative assessment of bone healing during osteogenic distraction, including conventional radiography and pixel value in digital radiology, ultrasonography, bone densitometry and scintigraphy, QCT, biomechanical evaluation, biochemical markers, and mathematical models. These methods complement each other in the monitoring of the bone-lengthening process, and all have advantages and disadvantages ( Table 2 ).

**Table 2:** Advantages and disadvantages of bone regenerate evaluation methods

Method	Benefits	Disadvantages
Plain X-ray	Simple, fast, and convenient	Limited sensitivity to early bone callus formation
	The most common method to assess bone healing	
Pixel value in digital radiology	Reliable, available, and cost-effective	Limited sensitivity to early bone callus formation
	Minimizes the variation in inter- and intraobserver responses observed with plain radiographs	
Ultrasound	Inexpensive, portable, ionizing radiation-free, enables early monitoring	Relatively limited sensitivity
		Limited limb-alignment assessment
		Limited evaluation of the final stages of osteogenic distraction
Bone densitometry by dual-energy X-ray absorptiometry	Gold standard for bone mineral density	High cost
	Indirect assessment of bone microstructure	Limited limb-alignment assessment
		Limited clinical application
Bone scintigraphy	Free of ionizing radiation	High cost
	Non-invasive method that enables the evaluation of changes in blood flow	Limited limb-alignment assessment
		Relatively low availability
Quantitative computed tomography	High precision and little error	High cost
	Directly related to histological specimens	High radiation dose
		Limited clinical application
Biomechanical evaluation	Direct assessment of bone quality	Limited clinical application
Biochemical markers	Theoretical basis to identify consolidation delay	Limited clinical application
Mathematical model	Theoretical non-invasive method to monitor osteogenic distraction	Limited clinical application

We believe that knowing the different methods currently available is fundamental. In addition, we understand that using several monitoring methods simultaneously may be an ideal solution, pointing to a future direction in the follow-up of osteogenic distraction.
