# Supplementary material for: Bone Regenerate Evaluation Methods
Source: Rev Bras Ortop (Sao Paulo). 2024 Mar 21;59(1):e1–9. doi: 10.1055/s-0043-1776021 (PMC10957264; doi:10.1055/s-0043-1776021)
Supplement: Related texts

# Research article: Métodos de avaliação do regenerado ósseo *

**Keywords:** avaliação quantitativa, distração osteogênica, fenômenos biomecânicos, fixação externa, radiografia

## Abstract

Desde que foi descrita por Ilizarov, a técnica de osteogênese por distração tem sido utilizada para o tratamento de diversas condições relacionadas ao trauma, infecções, tumores ósseos e doenças congênitas, na forma de transporte ou alongamento ósseo. Um dos dilemas mais comuns do cirurgião ortopédico que realiza distração osteogênica é o estabelecimento de um método reprodutível de verificação da progressão da osteogênese, que permita a detecção precoce de falhas no regenerado, para que se possa interferir de forma eficaz durante o tratamento, bem como determinar o tempo apropriado de remoção do fixador externo. Recentemente, vários métodos de monitoramento quantitativo, com os quais se poderia avaliar a recuperação da estrutura e as propriedades biomecânicas do regenerado ósseo em diferentes estágios, além do processo de cicatrização óssea, têm sido amplamente investigados. Por esses métodos, pode-se saber o conteúdo mineral ósseo, a densidade mineral óssea, a rigidez e o metabolismo ósseo. Nesta revisão, resumimos de forma abrangente as técnicas mais recentes para avaliar a cicatrização óssea durante a distração osteogênica, entre elas, métodos como a radiografia convencional e os valores de pixels em radiologia digital, a ultrassonografia, a densitometria e a cintilografia ósseas, a tomografia computadorizada quantitativa, a avaliação biomecânica, os marcadores bioquímicos e os modelos matemáticos. Consideramos fundamental o conhecimento dos diversos métodos à disposição atualmente e entendemos que a utilização de vários métodos de monitoramento simultaneamente possa ser uma solução ideal, que aponte para uma direção futura no seguimento da distração osteogênica.

## Introdução

Desde que foi descrita por Ilizarov, a técnica de osteogênese por distração tem sido utilizada para o tratamento de diversas condições relacionadas ao trauma, infecções, tumores ósseos e doenças congênitas, na forma de transporte ou alongamento ósseo. Para que esse procedimento tenha êxito, é essencial respeitar alguns fatores, como osteotomia de baixa energia, fixação estável, período de latência aceitável, ritmo de distração adequado e manutenção do membro funcional durante a distração. 1 A localização da osteotomia também influencia na qualidade do regenerado; as metafisárias têm uma melhor chance de levar à formação de calo ósseo suficiente. O período de latência entre a osteotomia e o início da distração varia entre cinco e sete dias, tempo necessário para permitir a formação e a organização do hematoma, para maximizar a formação do regenerado com vascularização adequada. A maioria dos autores recomenda uma taxa de distração de 0,25 mm 4 vezes ao dia. 1

Antes de iniciar o processo de distração osteogênica, são necessários uma anamnese e um exame físico criteriosos. É preciso identificar alguns fatores de risco que podem atrapalhar na formação do regenerado, tais como doenças concomitantes, uso de drogas e tabagismo, idade, desnutrição, diabetes mellitus, uso crônico de anti-inflamatórios não esteroidais, a etiologia da anormalidade e cirurgias prévias no local da osteotomia, tumores e alterações na vascularização dos tecidos circundantes. 1 2 3

Um dos dilemas mais comuns do cirurgião ortopédico que realiza distração osteogênica é o estabelecimento de um método reprodutível de verificação da progressão da osteogênese, que permita a detecção precoce de falhas no regenerado, para que se possa interferir de forma eficaz durante o tratamento, bem como determinar o tempo apropriado para a remoção do fixador externo. 4

## Avaliação e monitoramento do regenerado

## Radiografia

A formação do osso regenerado pode ser avaliada e monitorada de diversas formas, sendo a radiografia simples o método mais amplamente utilizado, 1 pois se trata do exame mais prontamente disponível e clinicamente mais acessível. 3 5 À vista disso, as radiografias são consideradas as imagens com melhor custo-benefício para acompanhamento de todos os aspectos da regeneração óssea; 6 7 em contrapartida, o método é dependente da experiência de quem o avalia. 1

Quando comparada a outros métodos, no acompanhamento dos pacientes em procedimentos de alongamento ósseo, a maior desvantagem da radiografia é o fato de que ela não é capaz de detectar a presença de novo regenerado até que uma quantidade considerável de cálcio tenha sido nele depositada. 3 A calcificação está biologicamente atrasada em relação à formação do osteoide, e algumas semanas podem se passar antes que uma indicação da resposta óssea ao alongamento seja identificada por meio da radiografia. 8

O sucesso da distração osteogênica depende da avaliação radiográfica sequencial, que é essencial para orientar decisões, como a remoção dos fixadores externos e a alteração da taxa de distração. A presença de três a quatro corticais cicatrizadas nas radiografias anteroposterior e lateral é comumente usada como ponto final para a cicatrização. 9 10 Embora útil, entre os profissionais há muita discordância com relação a este método, e há relatos de taxas de concordância entre observadores inferiores a 0,5. 11 A decisão de remover o fixador geralmente é tomada com base na radiografia simples e no exame clínico no momento da remoção do fixador; no entanto, usando esses critérios, taxas de fratura de 30% a 50% foram relatadas. 12 13 14 A corticalização adequada é definida como 2 mm de córtex visto com densidade semelhante ao osso normal e 3 desses córtices visíveis na radiografia. Em casos com alongamento de mais de 10 cm ou mais de 50% do comprimento original da tíbia, a presença de 3 córtices parece inadequada, e estender o tempo de remoção do fixador até que o quarto córtex também esteja bem formado é aconselhável para prevenir o retardo da subsidência da tíbia. 15

Shyam et al. 15 descreveram a razão de diâmetro do calo, calculada medindo-se a média dos diâmetros anteroposterior e lateral do calo e dividindo-a pela média dos diâmetros das extremidades proximal e distal da corticotomia. Mamada et al. 16 demonstraram um aumento significativo na taxa de fratura quando esta razão era inferior a 80%.

As técnicas radiográficas tradicionais permitem a avaliação qualitativa da neoformação óssea, mas isso nunca foi devidamente quantificado. Sem um método de medição objetivo, o erro intraobservador e interobservador pode ser alto em medidas subjetivas que usam radiografias. 17

Starr et al. 18 avaliaram o critério comumente citado, da presença de três das quatro corticais contínuas de pelo menos 2 mm de espessura nas radiografias anteroposterior e lateral, e encontraram valores baixos para os coeficientes kappa médios de confiabilidade relativos às respostas intraobservador (0,290) e interobservador (0,127), o que indica que a avaliação do número de córtices por si só não é um bomindicador do momento da remoção do fixador, sendo insuficiente como método isolado.

Eyres et al. 19 relatam que, embora a ultrassonografia e a densitometria óssea por absorciometria de raios-x de dupla energia ( *dual energy x-ray absorptiometry* , DEXA, em inglês) forneçam informações valiosas sobre a distribuição e a quantidade de osso novo formado durante a distração osteogênica, as radiografias com imagens de alta resolução foram úteis na detecção de pequenos defeitos corticais que não foram identificados pelas outras técnicas de imagem.

## Valores de Pixels em Radiologia Digital

As técnicas radiográficas tradicionais permitem a avaliação qualitativa da neoformação óssea, mas a sua quantificação não era possível até o desenvolvimento da radiologia digital. 8 20 Os métodos quantitativos incluem tomografia computadorizada quantitativa (TCQ), 21 22 cintilografia quantitativa com tecnécio 20 e densitometria óssea por DEXA. 19 Esses métodos medem a mineralização do osso regenerado, que se correlaciona com a sua rigidez, 23 mas essas investigações são custosas e requerem que o paciente seja submetido a exames de imagem adicionais. 24 A técnica do valor de pixel, usada em radiografias digitais, 14 25 mostrou-se um método econômico para medir as mudanças na mineralização do regenerado para fornecer parâmetros objetivos para a tomada de decisão. 17

Por meio do valor de pixel, avalia-se a densidade mineral óssea (DMO), e ele também pode ser usado para avaliar a cicatrização do osso regenerado, para comparar a densidade do osso regenerado com a do osso adjacente. À medida que a densidade do osso regenerado aumenta com a cicatrização, seu valor de pixel fica próximo ao do osso normal adjacente. 26 27

O valor de cinza (VC) é outro indicador usado para avaliar a cicatrização óssea. Uma imagem em tons de cinza é uma matriz de dados. Seu valor representa uma faixa específica de valores de brilho, na qual brilho 0 significa preto e brilho 255 se refere ao branco. A parte da imagem em tons de cinza com brilho intenso representa o objeto com alta densidade ou espessura; a parte com brilho tênue representa o objeto com baixa densidade ou espessura fina. 28

Singh et al. 6 avaliaram os valores de pixel do regenerado e dos segmentos ósseos adjacentes de forma seriada durante o alongamento ósseo de pacientes acondroplásicos. As razões de valor de pixel foram então calculadas (razão de pixel = [(valor médio de pixel do segmento proximal + valor médio de pixel do segmento distal)/2]/valor médio de pixel da formação de osso novo). Vários autores demonstram que pacientes que apresentam fraturas do regenerado têm relações de valor de pixel inferiores a 0,8 no momento da remoção do fixador. 5 14 15 17 As taxas de valor de pixel podem ser usadas como um suplemento para avaliação radiológica digital, e podem ajudar na detecção de distúrbios de cicatrização precoce e na personalização de suporte de peso. Também podem ser usadas como uma orientação objetiva para a remoção do fixador. Entretanto, este método não mede diretamente a rigidez do osso regenerado. 4 6

A técnica de valor de pixel com o uso de radiografias digitais minimiza a variação nas respostas inter e intraobservador que ocorre com as radiografias simples, 1 5 sendo considerada um método confiável, disponível e de baixo custo para avaliar a maturação do regenerado. 4 17 26 Todavia, por se tratar de método matemático com base em imagens obtidas por radiologia digital, a técnica de valor de pixel compartilha das limitações encontradas na radiologia convencional para avaliação do regenerado em suas fases iniciais. 26

## Ultrassonografia

O princípio do uso da ultrassonografia na avaliação do regenerado é o de que sinais de atenuação ultrassônica de banda larga podem ser empregados para avaliar a alteração da DMO. Quando o ultrassom passa pelo tecido corporal, ocorre a atenuação, cuja quantidade está relacionada às características do tecido. Usando a velocidade das ondas ultrassônicas e a atenuação da amplitude que passa pelo tecido ósseo, pode-se calcular a quantidade de conteúdo mineral ósseo (CMO), a estrutura óssea e a resistência óssea. 29 A avaliação do osso maduro normal por ultrassom é limitada pela incapacidade do ultrassom de penetrar na cortical óssea. No entanto, osso novo com remodelação incompleta e calcificação pode ser bem avaliado por ultrassom linear de alta resolução. 1 30 A ultrassonografia com contraste (USC) pode fornecer uma indicação precoce de neovascularização e uma base diagnóstica para a regeneração óssea deficiente. 31 A ultrassonografia também pode predizer a formação de calo ósseo ao revelar mudanças no fluxo sanguíneo ao redor do osso novo, o que pode compensar a má visualização precoce do calo na radiografia. 32

A ultrassonografia é um método não invasivo, eficaz, barato e livre de radiação ionizante para avaliar a cicatrização óssea, que pode detectar formação óssea nova quatro a seis semanas antes da radiografia, 26 além de indicar a taxa de formação de osso novo nos estágios iniciais de distração. 33 Portanto, a avaliação precoce é feita de forma melhor por ultrassom, que permite a detecção do osteoide não mineralizado e a presença de quaisquer defeitos no calo. 8

No entanto, Eyres et al. 19 observaram que a ultrassonografia não permite detectar alterações na região medular do osso após a ocorrência da corticalização, mesmo com o uso de sonda de frequência de 5 MHz, mas se mostra útil na identificação de defeitos na corticalização no regenerado que não foram reconhecidos por DEXA ou radiografia. Contudo, trata-se de método dependente do examinador, sujeito à experiência e à familiaridade do cirurgião com a técnica. Além disso, não permite a avaliação do alinhamento ósseo e apresenta limitações para avaliação dos estágios finais da distração osteogênica, tendo papel limitado na decisão de retirada do fixador externo. 26

## Densitometria óssea por DEXA

A densitometria óssea por DEXA usa fontes de radiação de raios X para emitir duas energias de radiação diferentes. Permite medir a sua absorção pelos ossos e tecidos moles separadamente, descartando a influência do tecido mole. Consequentemente, o CMO e a área de DMO (aDMO) podem ser medidos e as alterações nas trabéculas ósseas podem ser observadas. 34

A capacidade da densitometria óssea para determinar a quantidade e a taxa de formação de osso novo é uma vantagem sobre outros métodos como a ultrassonografia e a radiografia. O osso novo é identificado tanto por densitometria quanto por ultrassonografia dentro de uma a duas semanas após a osteotomia, e apenas após quatro a oito semanas por radiografia. O alinhamento e a distração dos membros podem ser medidos por densitometria óssea durante todo o período de alongamento, o que é uma vantagem em relação à ultrassonografia, que permite a avaliação do regenerado apenas durante os estágios iniciais da distração, quando ainda não ocorreu a corticalização. 19

Vários estudos 1 13 sugerem o uso da varredura DEXA como uma ferramenta na avaliação da qualidade do regenerado durante a fase de distração e na decisão sobre o momento adequado da remoção do fixador.

Saran e Hamdy 13 utilizaram a densitometria óssea em conjunto com a radiografia simples para determinar quando a DMO do regenerado se estabilizou, e encontraram uma taxa de fratura do osso regenerado após a retirada do fixador externo de 3,6% e taxa de deformidade de 0%, mesmo com a liberação de carga conforme tolerado e sem uso de imobilização pelos pacientes.

Shyam et al. 15 calcularam a proporção da DMO com base na relação entre as DMOs do osso regenerado e do osso normal, e observaram que um índice superior a 0,85 impediria significativamente a fratura do osso regenerado e a sua angulação após a remoção do fixador externo.

A medição da mineralização do córtex por densitometria óssea pode ser considerada um método objetivo de avaliação do regenerado, pois permite a sua avaliação quantitativa. Contudo, o seu alto custo e a sua baixa disponibilidade relativa limitam a sua aplicabilidade clínica em larga escala. 17 26

## Cintilografia Óssea

A cintilografia óssea trifásica é um método não invasivo para avaliar alterações no fluxo sanguíneo, a distribuição sanguínea e o metabolismo ósseo de forma semiquantitativa. Considera-se que o suprimento sanguíneo tem íntima relação com a capacidade de produção do regenerado na osteogênese por distração. 35

Kawano et al. 36 avaliaram se a cintilografia óssea com tecnécio poderia ser útil para a avaliação e a predição do regenerado ósseo, e compararam índices clínicos como os de distração, maturação e fixação externa com dados obtidos a partir da cintilografia óssea, como o índice de perfusão e as taxas de captação da imagem de equilíbrio e de captação da imagem tardia, e concluíram que a cintilografia óssea trifásica é uma modalidade confiável para avaliar a distração osteogênica em comparação com os índices clínicos, especialmente a taxa de captação da imagem tardia, que demonstrou a maior capacidade de predição.

Apesar do potencial preditivo da cintilografia óssea, há poucos estudos clínicos que a avaliam a sua utilização na distração osteogênica. 36 A cintilografia óssea apresenta como limitação o seu alto custo e a sua baixa disponibilidade relativa, o que dificulta sua aplicabilidade clínica em larga escala, além de não permitir uma avaliação concomitante do alinhamento ósseo.

## Tomografia Computadorizada Quantitativa

A TCQ é um método que permite medir a DMO e avaliar o alinhamento ósseo e a composição corporal por meio do uso de um software especial em um aparelho de TC. Este permite uma avaliação de alta precisão e pequeno erro, sendo um excelente método para medir alterações da DMO ao longo do tempo. 26

A TCQ se baseia nas diferenças de absorção de radiação ionizante por distintos tecidos, o que permite comparar as medidas de atenuação obtidas com os valores de referência padrão para calcular informações como o CMO e a DMO. 37 Além disso, imagens tridimensionais podem ser usadas na TCQ, o que permite a avaliação do calo ósseo, o que, por sua vez, viabiliza a realização de análises de elementos finitos para prever a força do calo ósseo, o que tem sido aplicado em pesquisas musculoesqueléticas. 26

Contudo, o alto custo e a dose alta de radiação devem ser considerados, além do fato de que sua aplicabilidade e disponibilidade não são suficientemente amplas atualmente. Mais estudos ainda são necessários para abordar essas questões. Com o desenvolvimento da TCQ, a avaliação do regenerado ósseo pode fornecer informações mais valiosas, como o monitoramento da cicatrização óssea e a previsão da força do novo osso por análises de elementos finitos. 26

## Avaliação biomecânica

Medir as mudanças nas propriedades mecânicas do osso é o método mais direto de se avaliar o processo de cicatrização óssea. A biomecânica óssea é baseada na teoria da mecânica de engenharia, que avalia a qualidade óssea pelas propriedades mecânicas do tecido ósseo sob ação externa e o efeito biológico do osso após estresse. 38 Os testes de flexão, torção, tensão e compressão são comumente usados para avaliar as propriedades mecânicas da distração osteogênica e do novo tecido ósseo. 39 40 Parâmetros mecânicos do osso, como rigidez à flexão e à torção, ajudam a entender a consolidação óssea. 41

A grande limitação da avaliação biomecânica no monitoramento do regenerado é o risco de possíveis danos que testes de estresse utilizados no processo de medição causariam ao osso, de modo que seu uso atualmente restringe-se à pesquisa médica. 26

Lineham et al. 42 descreveram a possibilidade de uma avaliação biomecânica indireta por meio da aferição da deflexão dos fios de Kirschner utilizados na montagem da fixação externa circular durante o alongamento e transporte ósseos. No seu conjunto de observações, os autores 42 constataram que a deflexão do fio esteve significativamente associada à estabilidade determinada clínica e radiologicamente. Embora se trate de um estudo piloto e o método não esteja disponível, o desenvolvimento de novos dispositivos de aferição biomecânica *in vivo* poderia ser útil à prática clínica.

## Marcadores bioquímicos

Teoricamente, a alteração do metabolismo ósseo pode levar a alterações morfológicas subsequentes. Em outras palavras, alterações em marcadores de remodelação óssea (MROs) deveriam ser mais precoces do que mudanças identificáveis na DMO. Portanto, os marcadores bioquímicos como os MROs são um potencial novo método de avaliação da cicatrização óssea, e podem ser um método complementar valioso aos exames de imagem. 26

Atualmente, vários tipos de RMOs foram identificados, 43 como a osteocalcina (OC), a fosfatase alcalina específica do osso (FAEO), o propeptídeo aminoterminal do prócolágeno tipo I ( *procollagen type I N-terminal propeptide* , PINP, em inglês) e o propeptídeo carboxiterminal do pró-colágeno tipo I ( *procollagen type I carboxy-terminal propeptide* , PICP, em inglês), que podem indicar as atividades biológicas de osteoblastos e osteoclastos *in vivo* . 44 45

Fink et al. 46 estudaram a relação entre RMOs e densidade radiográfica durante a distração osteogênica, e descobriram que a medição dos níveis séricos de OC e PICP pode trazer informações valiosas sobre a formação óssea durante o tratamento.

Leung et al. 47 estudaram um modelo de distração osteogênica em cabras, e encontraram uma forte correlação entre a atividade da FAEO no plasma e a morfologia radiológica e as propriedades biomecânicas do osso do neoformado. Isso mostra que podemos usar a FAEO para monitorar o processo de mudança e formação do calo ósseo.

Kumar et al. 45 estudaram prospectivamente 168 pacientes com fraturas fechadas da tíbia tratados com hastes intramedulares bloqueadas, e demonstraram que os marcadores de formação óssea (FAEO, OC e PINP) foram significativamente menores em pacientes com consolidação tardia.

Diversos marcadores metabólicos ósseos foram relatados para monitorar a cicatrização óssea, e alguns deles têm alto grau de viabilidade teórica. Entretanto, ainda são necessários mais estudos experimentais e clínicos bem desenhados para determinar a aplicabilidade clínica desses marcadores bioquímicos no acompanhamento e avaliação do regenerado ósseo. 26

## Modelo matemático

Reina-Romo et al. 48 apresentaram um modelo matemático baseado em uma estrutura de elementos finitos para estudar os padrões espaciais e temporais de distração osteogênica próximo ao local da osteotomia. Com uma taxa de distração de 0,3 mm por dia, há um aumento precoce na densidade óssea média; computacionalmente, essa taxa menor de distração é acompanhada por menor estímulo mecânico, o que resulta em uma osteogênese estimulada. Em contraste, uma taxa de distração de 2 mm por dia produz não união, 49 e isso está de acordo com a maioria dos resultados clínicos que consideram que uma taxa de distração de cerca de 1 mm por dia produz os melhores efeitos na regeneração tecidual. 50

Posteriormente, Reina-Romo et al. 51 estenderam o modelo de diferenciação previamente desenvolvido por meio da incorporação da assimetria tensão-compressão. O novo modelo considera que o modo de formação óssea em ambientes de tração compreenderia principalmente a ossificação intramembranosa, e sob cargas compressivas a ossificação endocondral seria o principal modo de ossificação. 52 Nesse sentido, o estímulo mecânico que ativaria a formação do tecido ósseo seria maior sob tensão do que sob compressão. 53

Com base nesses estudos, o modelo computacional biomecânico do processo de transporte ósseo baseado em modelos experimentais poderia vir a ser uma ferramenta útil no seguimento da distração osteogênica. 54

## Classificação do regenerado ósseo

Embora as radiografias possam fornecer informações valiosas sobre a taxa de distração e alinhamento do regenerado, a avaliação da qualidade e da quantidade desse novo osso deve ser feita com cuidado até que a confiabilidade e a significância dessas características sejam determinadas. 8

Na avaliação radiográfica, mudanças na posição do membro, na penetrância do feixe e na ampliação podem alterar significativamente a percepção da imagem obtida. As características sob escrutínio podem ser interpretadas de forma diferente entre os observadores, e a relação dessas características com o resultado não é clara. 8

Diversos autores tentaram classificar a regeneração óssea, mas, com algumas exceções, a confiabilidade e a reprodutibilidade desses sistemas de classificação não foram testadas. 2 8 55 56 Alguns desses estudos também têm a desvantagem de apresentar tamanhos de amostra relativamente pequenos e um número relativamente grande de fatores de influência que limitam a interpretação de seus achados. 49

A Tabela 1 mostra uma revisão dos sistemas de classificação do regenerado ósseo relatados em estudos com humanos e com animais.

**Tabela 1:** Visão geral dos sistemas de classificação do regenerado ósseo

Autor	Ano	Características
Catagni 55	1991	Normotrófico: primeiro osso radiodenso 20 dias após a corticotomia
		Hipertrófico: formação óssea antes de 20 dias ou osso mais largo do que as extremidades da osteotomia
		Hipotrófica: formação óssea retardada, após 30 dias, múltiplas radiolucências no regenerado ou osso em configuração de ampulheta
Hamanishi et al. 2	1992	1. Externo: regenerado fusiforme
		2. Reto: regenerado homogêneo tão largo quanto o osso original
		3. Atenuado: regenerado mais estreito do que o osso original
		4. Oposto: regenerado na extremidade oposta ao fixador
		5. Pilar: pouco regenerado, apenas na porção central
		6. Agenético: apenas calcificação esparsa no *gap* alongado
Orbay et al.	1992	Tipo I. Novo osso homogêneo unindo as duas extremidades da osteotomia
		Tipo II. A osteotomia é coberta por um segmento contínuo de osso novo, mas há uma descontinuidade em pelo menos uma de suas corticais, ou o osso tem aparência irregular
		Tipo III. Defeito radiolúcido completo em todo o local de formação de osso novo
Minty et al. 56	1994	1. Manchas ocasionais de osso novo
		2. Calo desorganizado
		3. Regenerado em camadas organizadas
		4. Corticalização precoce
		5. Ponte óssea completa ligando as duas extremidades da osteotomia
Donnan et al. 8	2002	Quanto ao formato do regenerado (fusiforme, contido, oposto ou atenuado)
		Quanto à polaridade do regenerado (polarizado ou não polarizado)
		Quanto à consistência do regenerado (homogêneo, lucente, estriado ou salpicado)
Li et al. 49	2006	Quanto à forma: baseada na largura do calo em comparação com o local ósseo original da osteotomia (fusiforme, cilíndrico, côncavo, lateral e central)
		Quanto ao tipo: baseado em quatro padrões de distração osteogênica (esparsa, homogênea, heterogênea e transparente) e três densidades (baixa, intermediária e normal)
Tirawanish e Eamsobhana 58	2018	Sistema que combinado o diâmetro e a densidade do regenerado ósseo (pontuação de 2 a 9)
		Parte I: diâmetro – porcentagem média dos diâmetros anteroposterior e lateral do regenerado em relação ao diâmetro ósseo no local da osteotomia (classificado em grupos de 1 a 5)
		Parte 2: densidade – subdividida em 1 (baixa densidade), 2 (densidade intermediária baixa), 3 (densidade intermediária) e 4 (alta densidade)

Catagni 55 descreveu sua classificação radiológica do regenerado durante a distração osteogênica com o aparelho de Ilizarov com base em uma experiência clínica de mais de 800 casos. Classificou os regenerados como normotróficos, hipertróficos e hipotróficos, e chamou atenção para a necessidade de monitoramento cuidadoso do regenerado ósseo em busca de características que possam influenciar o resultado final do alongamento. Embora esta classificação tenha fornecido uma visão importante sobre os problemas durante a osteogênese, foi baseada puramente na experiência de um observador, e não leva em consideração a variabilidade da resposta óssea devido à idade do paciente, ao local da osteotomia ou à patologia subjacente. 8 10 57

Donnan et al. 8 revisaram os sistemas de classificação existentes e combinaram as características essenciais das demais classificações em três grupos: forma, consistência e polaridade. Com base nesta nova classificação, observaram uma concordância interobservador moderadamente boa com relação à forma e à consistência, mas apenas razoável com relação à polaridade. Archer et al. 5 avaliaram a concordância inter e intraobservador para a classificação de Donnan et al., 8 e observaram uma confiabilidade interobservador moderada e uma boa confiabilidade intraobservador.

Li et al. 49 desenvolveram um sistema de classificação do regenerado ósseo com base na forma do calo radiográfico e no tipo de característica que ocorria em diferentes estágios durante o alongamento do membro desde a osteotomia, passando pela distração e consolidação, até a remoção do fixador. As características radiográficas da distração osteogênica foram classificadas quanto à forma e tipo. A forma foi baseada na largura do calo em comparação com o local ósseo original da osteotomia. O tipo foi baseado em quatro padrões de distração osteogênica (esparsa, homogênea, heterogênea e transparente) e três densidades (baixa, intermediária e normal).

O sistema de classificação de Li et al. 49 é considerado útil para registrar e monitorar a distração e consolidação do regenerado ósseo. 4 49 Devido à sua correlação confiável entre observadores e um alto nível de reprodutibilidade para observadores individuais, pode ser utilizada para o seguimento da distração osteogênica. 4 5 49

Isaac et al. 3 observaram, com base na classificação de Li et al., 49 que padrões de distração osteogênica homogêneos e heterogêneos dão bons resultados, enquanto os padrões transparentes e esparsos dão resultados ruins. Quanto à forma, observaram que a fusiforme, a cilíndrica e a lateral dão bons resultados, ao passo que a côncava dá um resultado ruim. Dessa forma, ao se deparar com esses padrões específicos, se é alertado sobre um possível resultado insatisfatório e, assim, pode-se planejar medidas de tratamento que anulem este resultado, como ajuste da taxa de distração, realização de distração-compressão ou enxerto ósseo.

Tirawanish e Eamsobhana 58 classificaram a densidade de calos em quatro padrões (três heterogêneos e um homogêneo). A ocorrência de padrões heterogêneos pode ser usada clinicamente para alertar o cirurgião sobre possíveis problemas, como velocidade de distração muito alta, instabilidade do fixador, diástase inicial do local da osteotomia ou correção de deformidade. Consideraram que o padrão ideal de cura era homogêneo, pois um padrão heterogêneo teria maior probabilidade de evoluir para um desfecho ruim. Esses autores 58 desenvolveram um sistema de pontuação aplicável à distração osteogênica em tratamentos de alongamento de membros inferiores, que é usado para registrar e resumir informações radiográficas, permite relacionar as características do calo ósseo, e tem o objetivo de prever resultados bons ou ruins. Assim, fornece um método de avaliação que pode ser usado para monitorar o progresso e prever possíveis problemas, o que permite o ajuste precoce do processo de tratamento, se necessário. Uma pontuação de 8 ou 9, por exemplo, seria indicativa de um bom resultado, enquanto uma pontuação inferior a 7 seria um indicador de um resultado ruim. Este sistema mostrou-se confiável e reprodutível por cirurgiões experientes e menos experientes.

## Considerações finais

Existem vários métodos para avaliar quantitativamente a cicatrização óssea durante a distração osteogênica, como a radiografia convencional e os valores de pixels em radiologia digital, a ultrassonografia, a densitometria e a cintilografia ósseas, a TCQ, a avaliação biomecânica, os marcadores bioquímicos, e os modelos matemáticos. Os diversos métodos descritos podem ser considerados complementares no acompanhamento do processo de alongamento ósseo, e cada um deles tem suas vantagens e desvantagens ( Tabela 2 ).

**Tabela 2:** Vantagens e desvantagens dos métodos de avaliação do regenerado ósseo

Método	Vantagens	Desvantagens
Radiografia simples	Simples, rápida e conveniente	Sensibilidade limitada para a formação precoce de calo ósseo
	Método mais comum de avaliação da consolidação óssea	
Valores de pixels em radiologia digital	Confiável, disponível e de baixo custo	Sensibilidade limitada para a formação precoce de calo ósseo
	Minimiza a variação nas respostas inter e intraobservador que ocorre com as radiografias simples	
Ultrassonografia	Barato, portátil, livre de radiação ionizante, e permite o monitoramento precoce	Sensibilidade relativamente limitada
		Limitação para a avaliação do alinhamento do membro
		Limitação para a avaliação dos estágios finais da distração osteogênica
Densitometria óssea por absorciometria de raios-x de dupla energia	Padrão de ouro para a densidade mineral óssea	Alto custo
	Avaliação indireta da microestrutura óssea	Limitação para a avaliação do alinhamento do membro
		Limitação a para aplicação clínica
Cintilografia óssea	Livre de radiação ionizante	Alto custo
	Método não invasivo que permite avaliar alterações no fluxo sanguíneo	Limitação para a avaliação do alinhamento do membro
		Baixa disponibilidade relativa
Tomografia computadorizada quantitativa	Alta precisão e poucos erros	Alto custo
	Diretamente relacionado com espécimes histológicos	Alta dose de radiação
		Limitação para a aplicação clínica
Avaliação biomecânica	Avaliação direta da qualidade óssea	Limitação para a aplicação clínica
Marcadores bioquímicos	Base teórica para a identificação de retardo de consolidação	Limitação para a aplicação clínica
Modelo matemático	Método não invasivo de base teórica para o seguimento da distração osteogênica	Limitação para a aplicação clínica

Consideramos fundamental o conhecimento dos diversos métodos à disposição atualmente, e entendemos que a utilização de vários métodos de monitoramento simultaneamente possa ser uma solução ideal, que aponte para uma direção futura no seguimento da distração osteogênica.
